# How Lovebirds Maneuver Rapidly Using Super-Fast Head Saccades and Image Feature Stabilization

**DOI:** 10.1371/journal.pone.0129287

**Published:** 2015-06-24

**Authors:** Daniel Kress, Evelien van Bokhorst, David Lentink

**Affiliations:** 1 Department of Mechanical Engineering, Stanford University, Stanford, California, United States of America; 2 Department of Mechanical Engineering and Aeronautics, City University London, London, United Kingdom; 3 Experimental Zoology Group, Wageningen University, Wageningen, The Netherlands; University of Alberta, CANADA

## Abstract

Diurnal flying animals such as birds depend primarily on vision to coordinate their flight path during goal-directed flight tasks. To extract the spatial structure of the surrounding environment, birds are thought to use retinal image motion (optical flow) that is primarily induced by motion of their head. It is unclear what gaze behaviors birds perform to support visuomotor control during rapid maneuvering flight in which they continuously switch between flight modes. To analyze this, we measured the gaze behavior of rapidly turning lovebirds in a goal-directed task: take-off and fly away from a perch, turn on a dime, and fly back and land on the same perch. High-speed flight recordings revealed that rapidly turning lovebirds perform a remarkable stereotypical gaze behavior with peak saccadic head turns up to 2700 degrees per second, as fast as insects, enabled by fast neck muscles. In between saccades, gaze orientation is held constant. By comparing saccade and wingbeat phase, we find that these super-fast saccades are coordinated with the downstroke when the lateral visual field is occluded by the wings. Lovebirds thus maximize visual perception by overlying behaviors that impair vision, which helps coordinate maneuvers. Before the turn, lovebirds keep a high contrast edge in their visual midline. Similarly, before landing, the lovebirds stabilize the center of the perch in their visual midline. The perch on which the birds land swings, like a branch in the wind, and we find that retinal size of the perch is the most parsimonious visual cue to initiate landing. Our observations show that rapidly maneuvering birds use precisely timed stereotypic gaze behaviors consisting of rapid head turns and frontal feature stabilization, which facilitates optical flow based flight control. Similar gaze behaviors have been reported for visually navigating humans. This finding can inspire more effective vision-based autopilots for drones.

## Introduction

When moving rapidly through a dense and cluttered environment like a forest, proximity information about tree trunks and branches is essential for negotiating a successful goal approach. To accomplish such navigational tasks, diurnal birds rely on vision. Remarkably, little is known about which visual cues birds actually use to orchestrate their rapid flight maneuvers during close range navigation in their habitat. Well-studied visual depth perception mechanisms for primate navigation and depth perception, in particular stereopsis, is very limited in birds and therefore not considered to play a role in rapid flight control [1]. This motivated studies to discover alternative gaze strategies birds might use. Recent findings indicate that flying birds sense retinal image motion, so called optic flow, to adjust their distance to walls, control their flight speed, stabilize position during hovering and negotiate landing on a goal location, such as a perch [2–6]. The way the bird’s eyes move through the environment defines how experienced optic image flow of objects like trees and branches is composed. During straight, translational flight optical image flow on the eyes is slow for distant objects and becomes faster as an object gets in closer proximity. Consequently, experienced translational optic flow can be used to obtain relative distance information [7]. In contrast, optic flow during a rotation is distant independent. Its magnitude depends only on the velocity of eye rotation in space and is equal for close and more distant objects [8]. Therefore, experienced rotational optic flow cannot be used to extract reliable distance information. To increase the efficiency of extracting depth information from optic flow, birds use rapid head saccades to separate short-duration rotational from long-duration translational motion, to compartmentalize eye motion in space [3].

Between rotational head saccades, head orientation is stabilized. Consequently, the time in which purely translational optic flow, and thereby proximity information, can be obtained is prolonged. Similar gaze behaviors have been reported for fast flying insects that rely on optic flow for depth perception [9]. Birds that were prevented from stabilizing their heads during flight inescapably crashed [10]. The findings that birds use optic flow to control flight were obtained by analyzing single modes of flight in isolation. Consequently, it is unknown how birds switch between gaze behaviors and optic flow usage when performing a complete sequence of navigational flight behaviors like take-off, turning and landing. Navigating humans for example tend to fixate the center of a goal that they want to approach while fixating the edges of an obstacle they want to avoid in order to improve optic flow based steering [11,12]. To what extent do navigating birds perform similar gaze patterns as insects or humans?

We addressed the question of how birds change their gaze between maneuvering tasks by analyzing the flight and gaze kinematics of lovebirds, a small generalist parrot, performing a rapid turn maneuver during a simple goal directed task. Wild lovebirds roost and forage in small flocks of 5–20 birds in the scrublands of central Africa and Madagascar. We investigated the subsequent flight behaviors: take-off and fly away from a perch, turn on a dime, and fly back and land on the same perch. Such a sequence resembles natural flight maneuvers regularly conducted by this social bird when competing for perch locations on trees or feeding spots. Based on the reports that about 85% of the total visually induced gaze shifts in birds are accomplished by head rotations [13,14] and eye movements are usually smaller than 10° in unrestrained birds [14–16], we approximated the lovebird’s gaze orientation during flight by tracking the head orientation in a defined visual environment. The resulting data enabled us to draw conclusions on conducted gaze behaviors and visual features used when maneuvering.

We find that maneuvering lovebirds conduct among the fastest gaze shifts so far reported for vertebrates. These shifts are preferably timed at specific phases within a wingbeat and, thus, are specifically adapted in their speed and occurrence to the requirements of visuomotor control of rapid flight.

## Methods

### Birds and training

**Fig 1:**
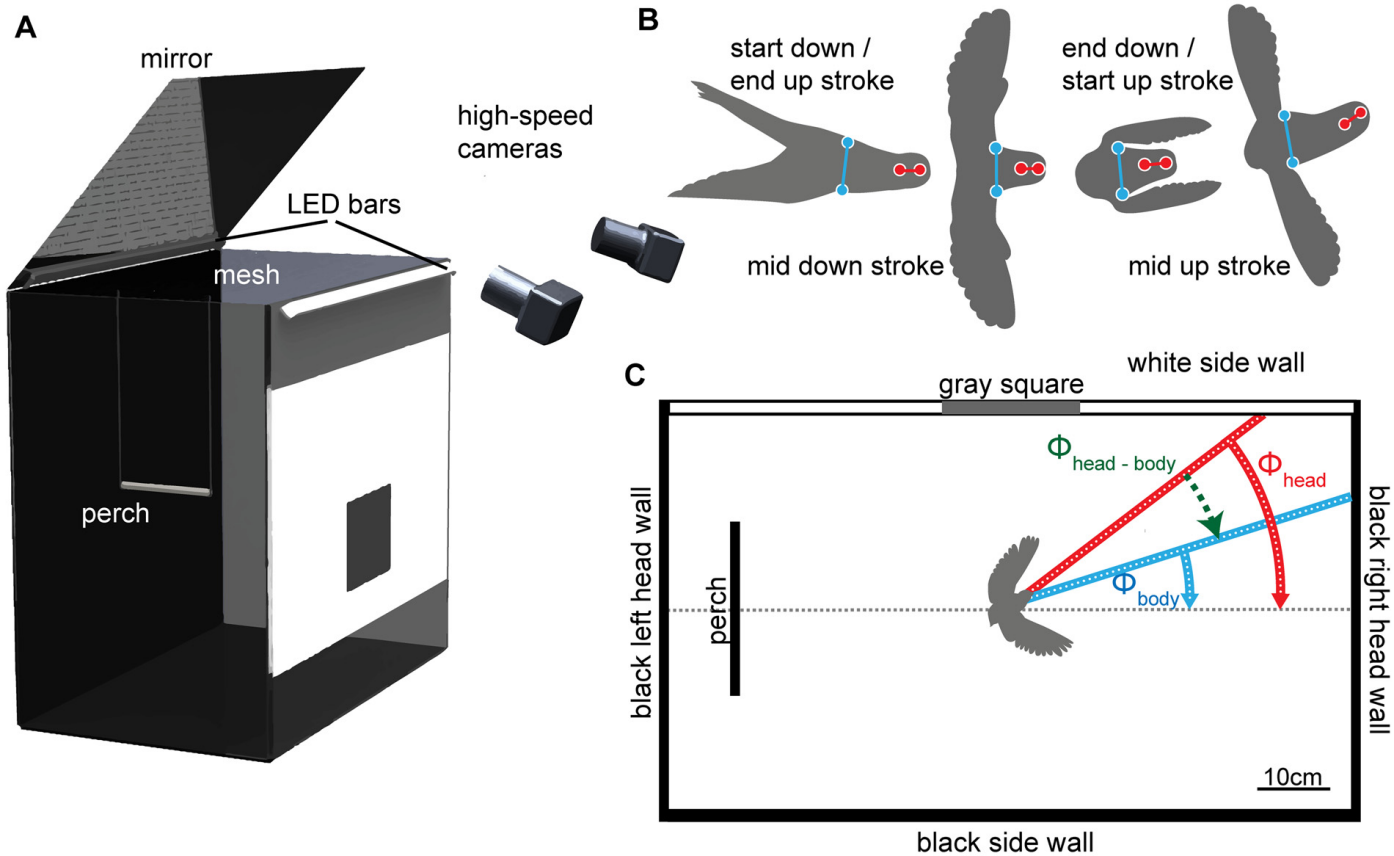
Experimental apparatus and analysis techniques. (A) Box-shaped flight arena in which lovebirds performed a U-turn flight maneuver starting and ending at the perch. The maneuver was filmed in stereo with two high-speed cameras at 2000 fps. (B) Illustration of the four time points within a wing beat in which we assessed the head and body orientation in “low resolution” with respect to wing beat phase. Red dots depict marker points on the head used to obtain its position and yaw orientation. Blue dots depict tracked shoulder positions used to obtain the yaw orientation of the body. (C) Schematic camera view into the arena. Note that due to the indirect view via the mirror above the arena, camera images were mirrored around the horizontal axis. To avoid confusion, we will refer to the flight scene as seen from the camera perspective. Red and blue lines show how yaw orientations of the head and body were obtained relative to a horizontal line in the image.

For flight recordings, we trained five lovebirds (*agapornis roseicollis*) to turn on a dime in our custom-built flight arena (Fig 1A). The first step was to train the birds to fly between two perches. In the second step, one perch was removed and birds were trained to fly away, turn and return to the remaining perch. During the third step, the width of the perch was decreased to about 21 cm, after which the birds were ready for the experiment. The birds were 2 years old at the time of the experiments and their weights ranged between 47 g and 56 g (2LG, ♀, 54.8 g; 2DG, ♀, 53.8 g; 1Y, ♀, 47.1 g; 3Y, ♀, 55.6 g; 3G, ♂, 47.4 g). The air temperature during the experiments ranged between 20.8 and 21.6°C. The birds were housed in pairs in enriched cages in which they received food and water *ad libitum*. For kinematic high-speed tracking, we painted waterproof ink marker points (edding 750 paint marker, edding International GmbH, Ahrensburg, Germany) on specific parts of the bird’s head (Fig 1B). Depending on the bird’s plumage color, we chose either white or black paint to maximize the image contrast of the applied marker points.

### Experimental setup and procedure

Flight experiments were conducted in a custom-built arena measuring 0.6 m x 1 m x 0.95 m (width x length x height; Fig 1A). The frame of the arena was made of aluminum and the wall panels were made of transparent LEXAN sheets. We closed the top with wire mesh (19 x 19 mm pitch, 1.4 mm thickness) to prevent birds from escaping. Two LED panels provided light from above; each panel held an array of five equally-spaced (~23 cm pitch) ultra-bright LEDs emitting 2650 lm ± 186 lm (BXRA-C2500, Bridgelux Inc., Livermore, CA, USA; Fig 1A). A 21.3 cm wide swinging perch was hung 11.5 cm away from the left side wall at a height of ~44 cm below the mesh. This perch, made of a 1.6 cm diameter PVC tube covered with sand paper, constitutes the bird’s only takeoff and landing place within the arena.

**Fig 2:**
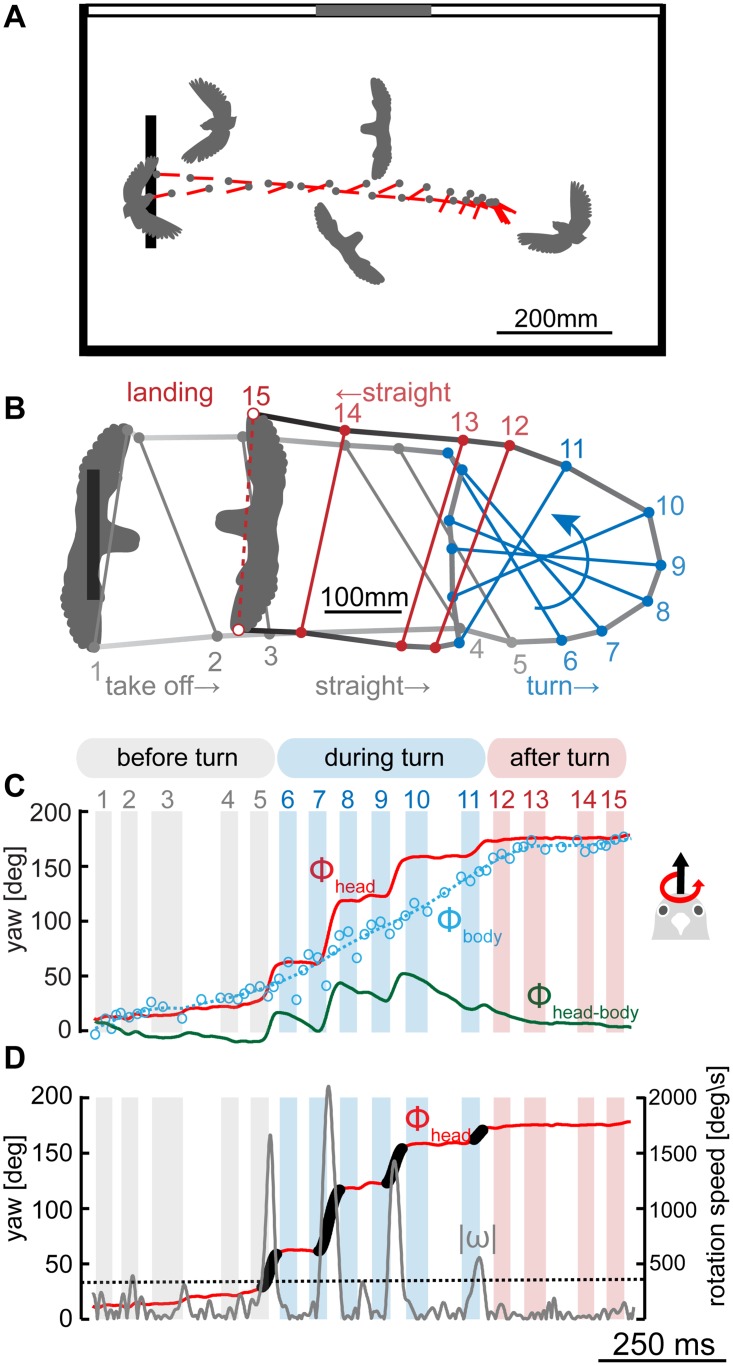
Individual example of a turning on a dime maneuver. (A) Head trajectory during a leftward U-turn flight maneuver in the flight arena. Plotted head position (gray dots) and head yaw orientation (red lines) are presented in 25 ms steps (50 frames). Because the turn is performed on a dime, traces after takeoff and before landing overlap. Arena wall grayscale (black, gray, and white) illustrates the inner wall texture seen by the bird. The thick black bar in the arena shows the perch position. (B) Top view of all 15 wing-tip positions at mid stroke during the same turn maneuver as shown in (A). Deflections in wing tip traces (wing beat 5–6 & 11–12) were used to separate the continuous flight maneuver into three consecutive phases: before turn (gray), during turn (blue) and after turn (red). (C) Head (red) and body (blue) yaw orientation angle (Φ) during a left turn maneuver. Φ is calculated in relation to a horizontal axis in the flight arena (see Fig 1 and methods). Φ values of 0° indicate an orientation along the horizontal axis facing away from the perch whereas Φ of 180° indicates an orientation along this axis facing towards the perch. Positive Φ deflections indicate a turn to the left, negative ones a turn to the right. The Φ difference angle between head and body is shown in green. Body data was filtered and interpolated (blue dashed line, see methods) to calculate the Φ head-body angle. Vertical bars represent the downstroke phases and thereby the wing beat timing before, during and after the turn. (D) Saccade detection (black, see text) based on absolute yaw rotation velocity (ω, gray graph). The dashed gray line illustrates the saccade detection threshold of 400°/s that ω had to exceed for at least 12 ms (24 data points) to define a head turn as saccadic.

One of the arena’s inner sidewalls was covered with a white cardboard sheet over the full length. This white wall had a small grey textured rectangle measuring 25 cm x 20 cm positioned at its center (Fig 1A). The arena floor was covered with plastic film to increase the birds’ contrast on the video images. As the illuminated flight arena was placed in a completely darkened room, all its walls except one sidewall appeared black to birds within it. Consequently, wall edges between the white and the dark walls constitute features with strong contrast within the arena. To film the flying bird from above, two laterally placed high-speed cameras (Photron APX and PCI camera, Photron Inc, San Diego, CA, USA) were directed at an inclined mirror above the arena (Fig 1A). For orientation reference: in the following sections, we will describe our findings based on the recorded camera images which are mirrored around the horizontal axis—thus right and left arena sides are switched. Consequently, when mentioning the white sidewall appearing left in our figures (Figs 1C and 2A), we are actually describing the right arena sidewall (see Fig 1A & 1C for clarification). The same holds for turning directions (S1 Movie).

To initiate the turn on a dime flight, the light was turned on and a hand wave cue was given when the bird was adjusted to the light level. Lights in the flight arena were turned off in between recordings to let the bird rest and discourage preening. During this time, the recorded flight videos from the two high-speed cameras were stored to disk.

### Video analysis

#### Low resolution wingbeat related tracking & 3D marker reconstruction

Flight behaviors were filmed with two synchronized cameras at 2000 frames per second and a resolution of 1024 x 512 pixels (S1 Movie). To obtain the marker points’ 3D position in the filmed volume, we used a stereo triangulation procedure. Marker tracking and 3D marker reconstruction was done with the “DLTdv5” toolbox [17] for MATLAB (The MathWorks, Natick, MA, USA). 3D camera calibrations were created with the “easyWand” toolbox for MATLAB [18]. Each flight recording lasted about two seconds, which resulted in about 4000 recorded frames per camera and flight. As the manual marker tracking would have taken a single person about four years to complete for all recordings, we had to reduce the tracking procedure in a reasonable way. Bird flight dynamics depend on the kinematics of their wingbeat [10,17,19,20]. Therefore, we decided to track head marker points and shoulder positions only at four specific instances within a wingbeat, which enabled us to perform wingbeat related tracking. A complete wingbeat is defined by a complete down- and an upstroke of the wings. We noted the start and end times of a single wingbeat manually by going through the flight recordings frame by frame. We marked a frame as start of the downstroke (end of the upstroke) when the wings were in their most dorsal position. Similarly, we marked a frame as end of the downstroke (start of the upstroke) when the wings were in their most ventral position. Beside the start and end points, we also marked the two frames half way through an up and downstroke, respectively (Fig 1B). Maneuvering lovebirds changed their wing beat frequency throughout the recorded flight. Consequently, the wingbeat related tracking frequency varied as well and ranged from about 35 to 70Hz (four times the wingbeat frequency).

#### High resolution tracking

One of our main interests in this study was to analyze the gaze behavior of maneuvering birds. The temporal resolution of the wingbeat related tracking procedure was not sufficient to analyze head and thereby gaze kinematics in detail. Therefore, we analyzed head data in multiple flights (at least three per bird), for each bird at full temporal resolution of 2000 Hz. The selection of flights was made based on following criteria: (i) the birds had to be in the camera view from beginning to the end of the flight. (ii) The flight contained not more than one complete U-turn. (iii) Changes in its flight altitude were less than 21 cm. (iv) Birds were facing into the flight direction (± 45°) before taking off. (v) Landing on the perch was successful on the first attempt, and to optimize marker tracking, (vi) head makers were not covered by the wires of the top mesh for long flight passages. 16 flight recordings of five birds fulfilled these criteria. Automated marker tracking for these recording was done with the open source software “ivTrace” (http://opensource.cit-ec.de/projects/ivtools) [21–23]. We call the tracking procedure at full temporal resolution “high resolution tracking”.

#### Data filtering

Due to marker coverage by the wires of the top mesh and due to varying marker contrast levels for the differently colored birds, tracking could not be done completely automatically but was interspersed by sections in which we tracked manually. To account for noise introduced by our tracking procedures and digital jitter, we filtered the obtained marker point coordinates and obtained yaw orientations with a penalized least-squares smoothing algorithm based on a Whittaker filter [24,25]. This filter algorithm is in its function similar to a Savitzky-Golay filter but much more resource efficient [24]. The smoothing parameter λ was conservatively chosen to result in cross-validation errors (cve) to the original data below two (λ_coordinates_ = 100, cve_coordinates_ < 0.5; λ_head_ = 5000, cve_head_ < 1; λ_body_ = 1000000, cve_body_ < 2).

After 3D reconstruction of marker point and shoulder positions, we calculated the head and body yaw orientations for each time step. Their yaw orientation Φ was defined as the arctangent of the vector connecting both head makers (Φ_head_) and of the normal of the vector connecting the birds’ shoulders (Φ_body_) (Fig 1C). As body data was obtained at the lower, wing beat related temporal resolution, we filtered it with a Whittaker filter (see methods) and interpolated the data up to 2000 Hz to obtain a highly resolved Φ difference angle between head and body. The total error in 3D position estimation was on average 1.3 mm ± 0.4 mm. This value was based on the 3D reconstruction and calibration errors averaged across all tracked points and birds. The resulting orientation error is about 3.8°.

Similar to earlier studies addressing rotational head movements in free behaving animals [3,21], we defined head yaw saccades based on rotations exceeding a threshold speed for a certain minimal duration. Rotation velocities (ω) were obtained by taking the derivative from the filtered yaw orientation data. For our data, a rotational velocity of 400°/s and a duration of 12 ms (i.e. 24 consecutive frames) turned out to reliably detect head saccades. By comparing the onset of rotational velocity with the point in time when it exceeded 400°/s, we derived that saccade durations had an uncertainty of about 4 ms.

#### Data sets for the quantitative data analysis

The quantitative saccade and landing analyses were based on high-resolution tracking data (2000 Hz) consisting of 3–4 flights for each of the five tested birds (see classification criteria above). To avoid strong effects of ascending or descending maneuvers and have better comparable flights, we only analyzed turn flights for the wingbeat analysis where height changes during the turn were minimal (< 21 cm) resulting in 9–10 flights per bird. Quantitative analysis of gaze orientation was done first with the smaller high-resolution data set, and after validating that found distributions are qualitatively similar, the analysis was extended to the larger low-resolution wingbeat related data set that included all above mentioned flights. This set contained 12–26 flights per birds. In all analyses, we averaged the results per bird before comparing or averaging across birds.

#### Analysis of wingbeat and stroke period distributions

Wingbeat and downstroke / upstroke periods were analyzed for bimodal distributions using the Gaussian mixture models algorithm (GMM) of the MATLAB statistics toolbox. The GMM algorithm assumes that the wing data are a mixture distribution, where the probability density function is a combination with coefficients that sum to 1 (*ξ*_*1*_ *+ ξ*_*2*_ *=* 1),

f(x)=ξ1N1(μ1,σ1)+ξ2N2(μ2,σ2), 1

where μ_1_ and μ_2_ are the distribution means, σ_1_ and σ_2_ the standard deviations and ξ_1_ and ξ_2_ the mode weights. A fitted distribution was categorized as bimodal if the following conditions were true:

|μ1−μ|>2max(σ1,σ2)

and when

min(ξ1;ξ2)>0.1

#### Advance ratio

The advance ratio J was calculated for each wingbeat:

$$J=\frac{V}{2AfR},$$ 2

where *V* is the wingbeat averaged flight speed in m/s obtained from taking the derivative of head position data, *A* is the wingbeat amplitude in radians obtained from calculating the angles between most dorsal and most ventral wing tip position divided by 2*f*, the wingbeat frequency in Hz and *R*, the root-to-tip wing length [26]. The root-to-tip radii ranged between 12.62 cm to 14.03 cm for the five tested birds.

### Ethics statement

All training and experimental procedures were approved by the Institutional Animal Care and Use Committee of Wageningen University (DEC; Dierexperimentencommissie; protocol 2011095.b; in accordance with 86/609/EEC). Birds were housed in pairs in enriched cages, which included toys and artificial full-spectrum lights.

## Results

To investigate the saccadic gaze behavior and visual cues that maneuvering lovebirds use, we analyzed turning flights of five individuals. In all recordings, we observed a very stereotypical turning behavior in which the head initiated the turn with saccades, while the body followed with a slower but more constant turning behavior. Remarkably, in 90% of the analyzed flights (*n* = 92 flights, *N* = 5 birds) lovebirds turned left towards the white sidewall. Only 10% of the analyzed flights contained right turns towards the dark sidewall (*n* = 10 flights, *N* = 3 birds, see wall configuration in Fig 1C). Apparently, lovebirds preferred to turn towards high contrast features in the arena when performing a U-turn maneuver. Therefore, we will focus our quantitative analysis on left turning flights for which we obtained a more representative sample size.

**Fig 3:**
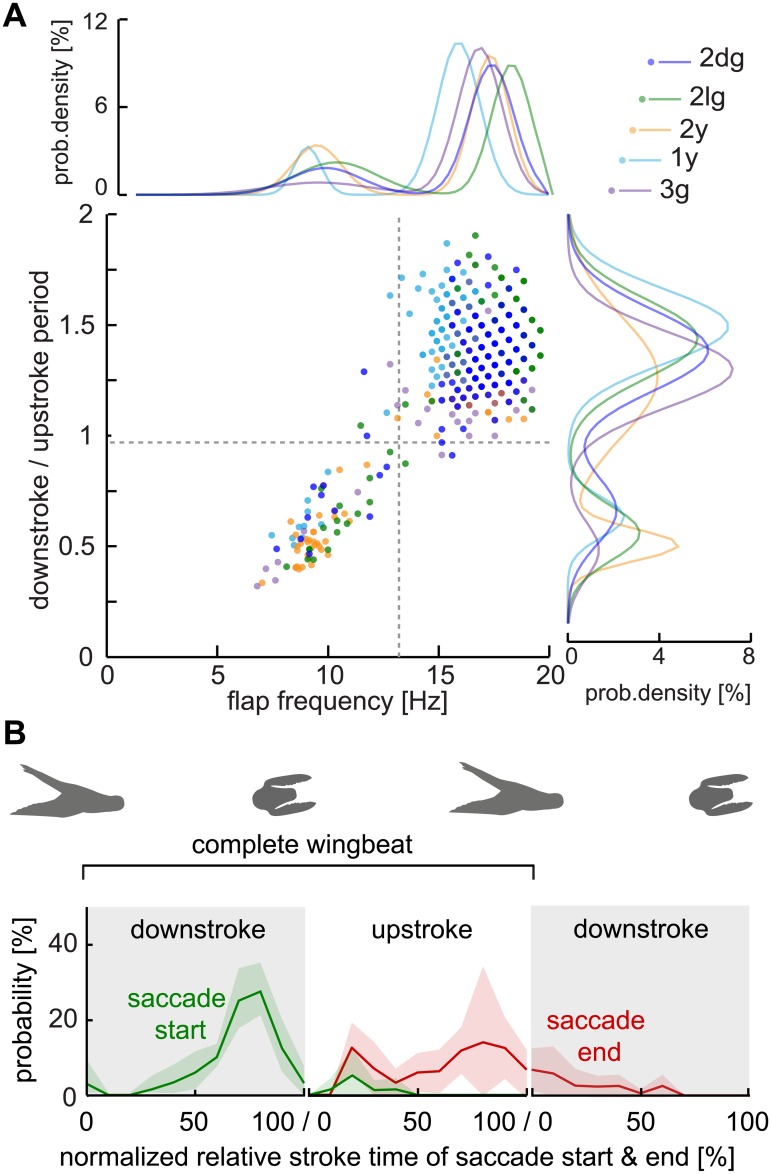
Lovebirds performed an intermittent flight style with two wingbeat distributions in which saccades are started during downstrokes. (A) The downstroke / upstroke phase ratio *vs*. instantaneous flap frequency distribution for individual wingbeats of five birds. A phase ratio of 1 indicates up- and downstrokes of equal duration, values <1 indicate longer upstrokes, values >1 longer downstrokes. Normalized bimodal Gaussian fits are shown for flap frequency (top) and for downstroke / upstroke time ratios (right). The bird-specific bimodal distribution parameters for the flapping frequency are: 2dg: *μ*_*1*_ = 9.78, *σ*_*1*_ *= 1*.*61*, *μ*_*2*_ = 17.26, *σ*_*2*_ *= 1*.*01*; 2lg: *μ*_*1*_ = 10.26, *σ*_*1*_ *= 1*.*83*, *μ*_*2*_ = 18.14, *σ*_*2*_ *= 0*.*91*; 2y: *μ*_*1*_ = 9.39, *σ*_*1*_ *= 1*.*1*, *μ*_*2*_ = 17.19, *σ*_*2*_ *= 0*.*86*; 1y: *μ*_*1*_ = 8.97, *σ*_*1*_ *= 0*.*6*, *μ*_*2*_ = 15.76, *σ*_*2*_ *= 0*.*96*; 3g: *μ*_*1*_ = 9.49, *σ*_*1*_ *= 2*.*3*, *μ*_*2*_ = 16.72, *σ*_*2*_ *= 1*; For downstroke / upstroke periods the obtained bimodal distribution parameters are: 2dg: *μ*_*1*_ = 0.5, *σ*_*1*_ = 0.07, *μ*_*2*_ = 1.26, *σ*_*2*_ = 0.27; 2lg: *μ*_*1*_ = 0.56, *σ*_*1*_ = 0.13, *μ*_*2*_ = 1.43, *σ*_*2*_ = 0.17; 2y: *μ*_*1*_ = 0.48, *σ*_*1*_ = 0.07, *μ*_*2*_ = 1.26, *σ*_*2*_ = 0.27; 1y: *μ*_*1*_ = 0.62, *σ*_*1*_ = 0.09, *μ*_*2*_ = 1.49, *σ*_*2*_ = 0.17; 3g: *μ*_*1*_ = 0.48, *σ*_*1*_ = 0.12, *μ*_*2*_ = 1.3, *σ*_*2*_ = 0.17. The horizontal gray line separates the bimodal distributions at a downstroke / upstroke ratio of 0.94 (average midpoint between bimodal distribution peaks among birds). The vertical gray line separates the bimodal distribution at a flap frequency of 13.3 Hz (average among birds); *n* = 697 wing beats, *N* = 5 birds. Due to the 2000 fps sample frequency, and the fact that wingbeat, downstroke, and upstroke time are all integer values measured in number of frames, the data appear in a raster and can overlap precisely among wings beats, flights and birds. (B) The normalized saccade distributions illustrate when a saccade was started and ended during the downstroke *vs*. the upstroke phase. Shown is the average across birds (solid lines) and the standard deviation (shaded area). Binning: 0:10:100; *n* = 72 saccades, *N* = 5 birds.

**Fig 4:**
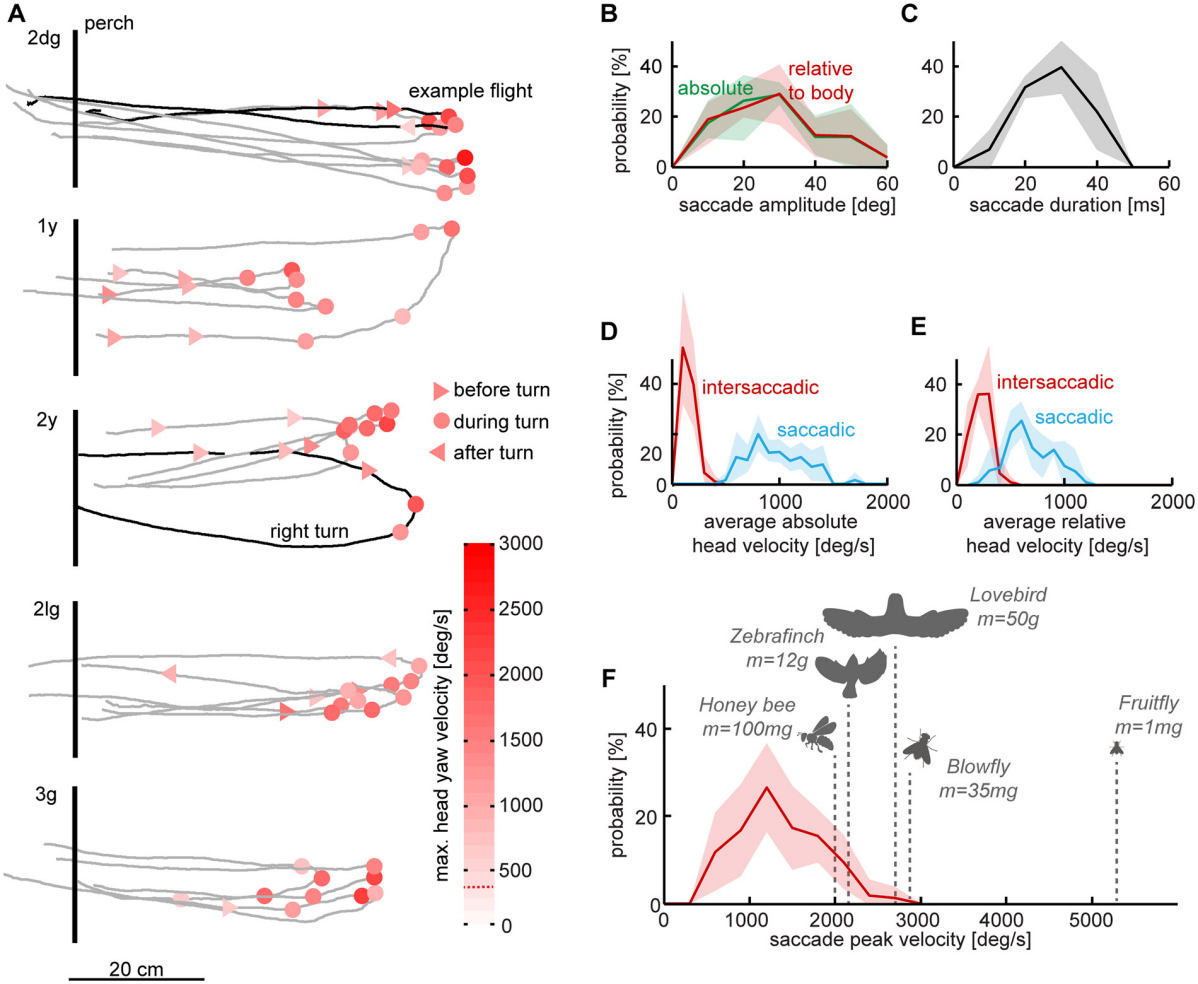
Head saccades occur predominantly during the turn and their speed compares to insects. (A) Flight traces of all birds depicting the position of saccade initiations during a U-turn. Turning flight traces are shown as grey lines, saccades as red colored symbols: triangles depict saccades made before or after the turning phase, cycles illustrate saccades during the turning phase. The dashed red line in the color bar represents the saccade detection threshold of 400°/s. The perch position is represented by the vertical black bar. A rare right turn flight of bird 2y is illustrated for representative reasons by the thicker line, n = 16 flights, N = 5 birds. Panels (B-F) illustrate differing head kinematics during intersaccades and saccades as well as the extraordinarily fast nature of lovebird head saccades. The shaded areas illustrate the standard deviation between birds. (B) Amplitudes of horizontal head saccades. Shown are the normalized absolute saccade amplitude distributions relative to a horizontal axis through the flight arena and the normalized relative saccade amplitude relative to the body yaw orientation. Binning = 0:10:60. (C) Normalized average of saccade duration across birds. Binning = 0:10:50. (D) Normalized average head yaw rotation speed in space for saccades and phases between saccades (intersaccades). Binning = 0:100:2000. (E) Normalized average head yaw rotation speed relative to the turning body, plotted for both saccades and intersaccades. Binning = 0:100:2000 (see text for definition). **(F)** Peak saccadic rotation speeds of the head. To compare the performance of lovebirds to other flying animals showing similar saccadic gaze behaviors, we inserted reported saccadic peak rotation speeds of the honey bee [28], zebra finch [3], the blowfly [27] and the fruitfly [29]. Binning = 0:300:3000. *n* = 72 saccades, *N* = 5. Saccade analysis is based on video data resolved at 2000 Hz.

**Fig 5:**
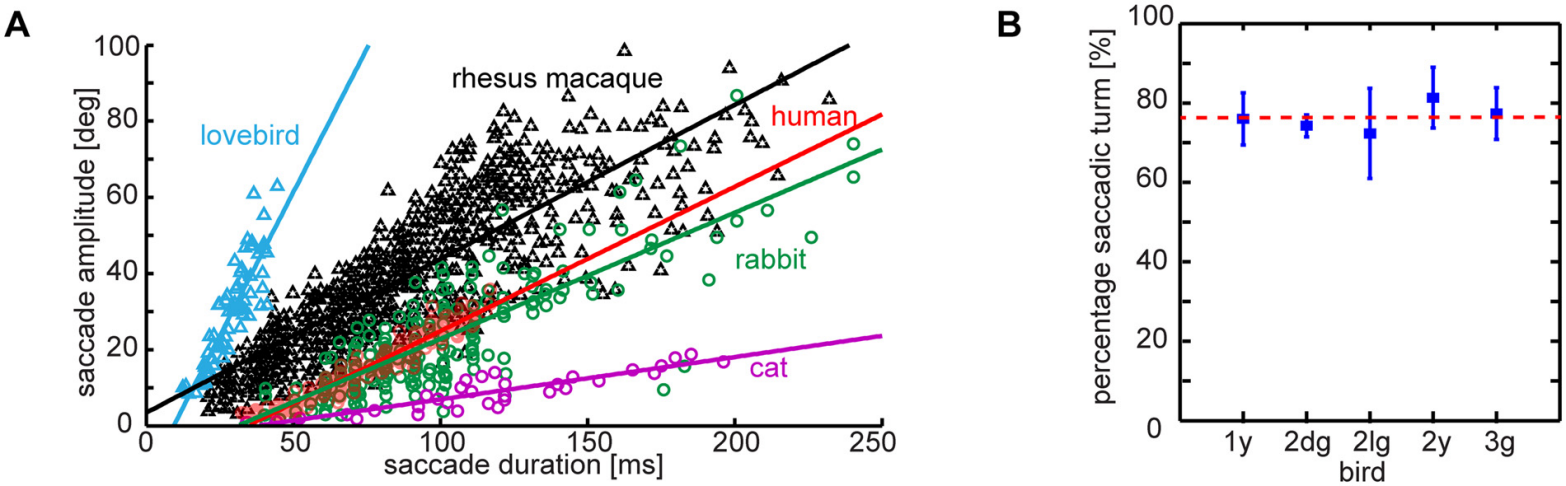
Lovebird head rotation is predominantly saccadic at the highest average speed recorded amongst vertebrates. (A) Comparison of horizontal saccade amplitude as a function of its duration. Shown are saccade amplitudes and durations measured in this study (blue triangles, *n* = 72, *N* = 5) and data for other species extracted from earlier publications. Gray triangle markers represent the combined eye-head gaze shifts of rhesus macaques (*n* = 544, *N* = 2) [30]. Red, green and violet circles illustrate horizontal eye saccades in humans (*n* = 187, *N* = 3) [31], rabbits (*n* = 191, *N* = 2) [33] and cats (*n* = 34, *N* = 2) [32]. We coarsely approximate average head rotation velocities by fitting the data with a linear regression. Line equations: lovebird: y = 1.5*x-14, slope = 1500°/s, R^2^ = 0.78; rhesus macaque y = 0.4*x+3.4, slope = 400°/s, R^2^ = 0.69; human: 0.38*x+3.4, slope = 380°/s, R^2^ = 0.93; rabbit: y = 0.33*x-10, slope = 260°/s, R^2^ = 0.67, cat: y = 0.11*x-4.2, slope = 110°/s, R^2^ = 0.81. (B) Proportion of saccadic turns on the whole U-turn maneuver. Shown is the average cumulative saccade amplitude (and standard deviation) as a percentage of the whole turn amplitude, with the red line showing the average across birds (*n* = 72 saccades, *N* = 5 birds).

**Fig 6:**
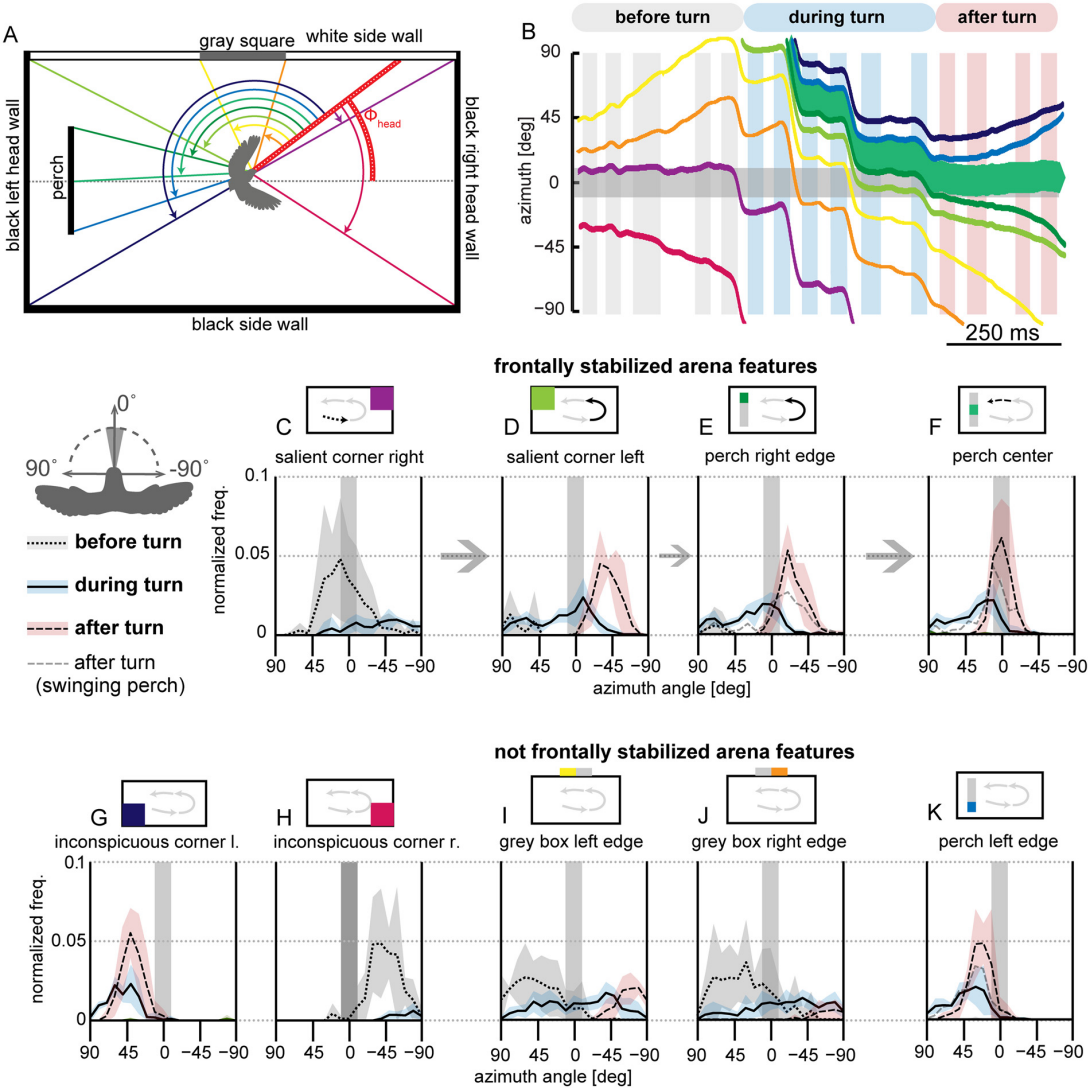
Distributions of intersaccadic azimuthal feature positions reveal that maneuvering birds stabilize arena features in their frontal visual field. (A) Schematic top view into the arena. Azimuthal positions of wall corners, the gray square and the perch (colored lines) were obtained relative to the bird’s head yaw orientation (red line). (B) Relative azimuthal angles of arena features for the example flight shown in Fig 2. The thick green line depicts the angle of the perch center. The gray shaded area represents feasible horizontal eye motions of ±10° relative to the horizontal head orientation. Positive azimuthal angles represent the left visual hemisphere, negative values the right visual hemisphere (see bird illustration above legend). Note that the diverging azimuthal angles of the perch edges (dark green and blue lines) are caused by the bird getting closer to the perch. Approaching the perch causes the retinal size of the perch to expand in the bird’s frontal visual field. (C-F) Averaged relative azimuthal distributions of arena features that were stabilized in the frontal visual field in the intersaccadic phases during a turning on a dime maneuver; before the turn (C: fine dashed line), during the turn (D & E: solid line) and after turning (F: coarse dashed line). Averaged distributions illustrate left turn flights (*n* = 92, *N* = 5). Standard deviations across birds are illustrated by the colored areas. (G-K) Intersaccadic azimuthal distributions for arena features that were not stabilized in the frontal visual field. By stabilizing the perch center frontally after the turn (F), the right and left edge distributions are positioned more laterally, are broader and have lower peaks than the center (E & K). The vertical bar extending ±10° illustrates feasible horizontal eye motions relative to the head orientation in unrestricted birds (review: [14]). Perch position for 92 flights is approximated by using the position of a static perch (thick lines in panel E-F, *n* = 92 flights, *N* = 5). For 15 left-turn-flights tracked at 2000 Hz, the position of the swinging perch relative to the birds was tracked as well (gray dashed line in panel E, F & K *n* = 15 flights, *N* = 5). For normalization, we divided each distribution by the cumulative sum of all other feature distributions. Binning = -90:10:90.

**Fig 7:**
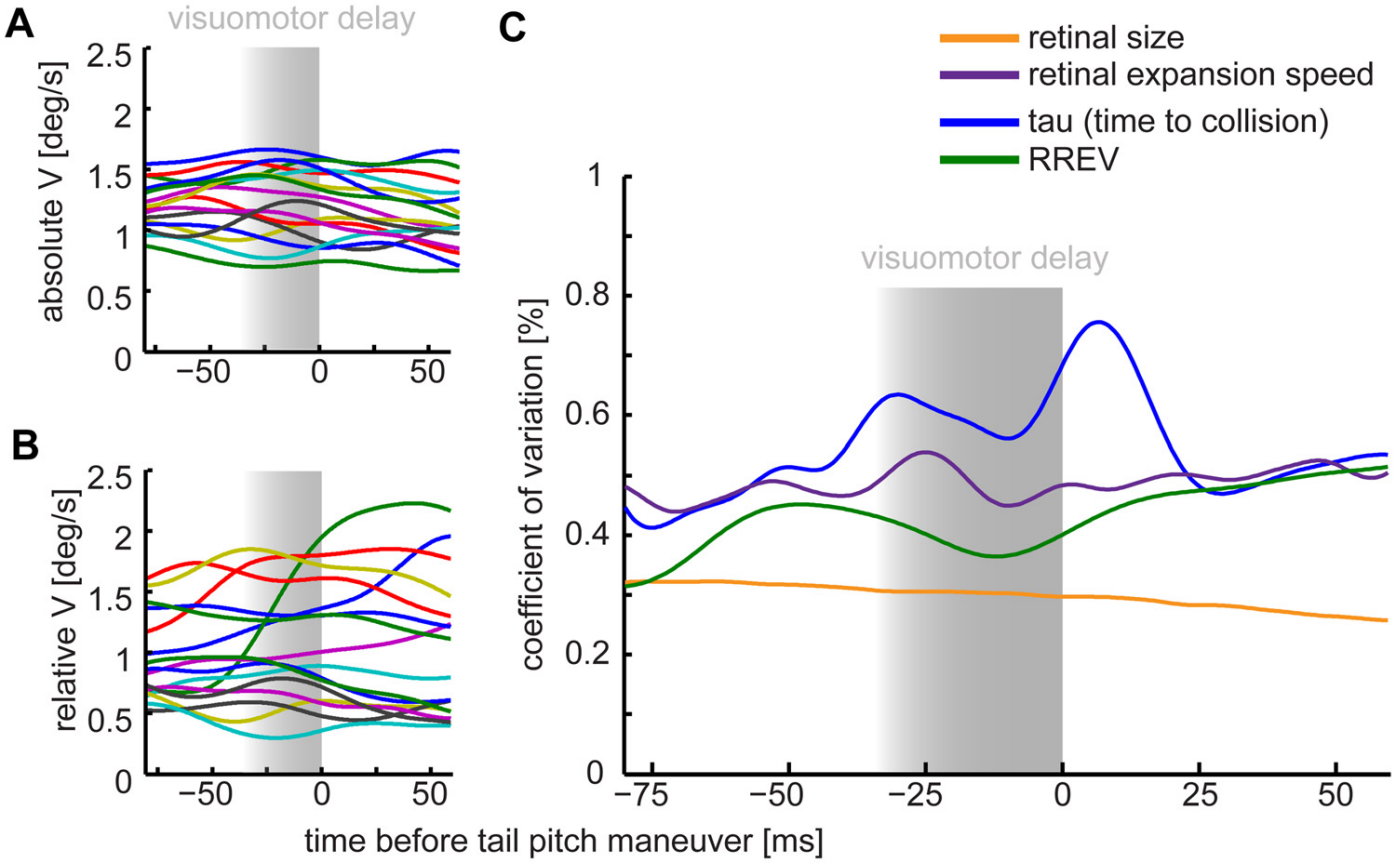
Low variation of retinal size and relative expansion velocity (RREV) of the approached perch suggests these cues matter for controlled landings on a swinging perch. We defined the tail pitch as the behavioral indicator for landing initiation (time = 0 ms). Negative time values represent the time before and positive time values the time after the downward pitch of the tail feathers. Shaded areas ranging from -30 ms to 0 ms mark the minimal time period of visuomotor delay during which visual flight control is unlikely. Absolute horizontal flight speed (A) has less variation across flights than relative horizontal flight speed (B) with respect to the moving perch. (C) The most parsimonious landing parameters are indicated by a minimum in the coefficient of variation (c.v.) across flights and birds. The retinal size (orange) and RREV (green) for the approached perch varied less that the parameter tau and the retinal expansion. Tail pitch timing was extracted individually from high-speed flight videos. *n* = 16, *N* = 5 birds.

To give insight into how an actual recorded U-turn flight looks, we will illustrate one example first (Fig 2, S1 Movie) before presenting quantitative results across birds (Figs 3 to 7). In a typical recorded flight, the lovebird took off from the perch, flew more than halfway into the arena, performed a rapid turning maneuver ‘on a dime’ in which it oriented itself back to the perch, flew back to the perch and landed on it (Fig 2A).

We divided the U-turn flight into three segments to analyze each phase individually: (i) the straight flight phase after take-off and before the turning maneuver (before turn), (ii) the turning phase (during turn) and (iii) the straight flight phase after the turn until landing (after turn). The segmentation was based on the position of the wingtips during mid-stroke. When the turn was initiated, the tip position changed characteristically by deviating from its previously relative straight trace (Fig 2B: transition gray to blue bars). The phase after turning started when the wingtip positions were again aligned along a straight trace (Fig 2B: transition blue to red phase). Throughout the recorded flights, wingbeat duration varied as indicated by the differences in down- and upstroke time (Fig 2C, S1 Movie), showing that lovebirds perform intermitted flight.

Maneuvering lovebirds had a relative constant head orientation before and after the turn (Fig 2C, gray and red bars). However, during the turn the birds changed their head orientation in a saccadic fashion; during short bursts they turned their head very quickly into the turning direction while keeping head orientation stable in between (Fig 2C, blue phase). Head saccades were typically faster than 1000°/s, and higher speeds beyond 2000°/s were also observed, as illustrated by the second saccade in Fig 2D. While we could track head data automatically at 2000 fps, we obtained body data manually at four times the wingbeat frequency (at about 68 Hz; see methods), and fitted them with a penalized least-squares smoothing algorithm for non-continuous data [24]. In contrast to the head, the body turned smoother and slower during the turn, following the head without saccades (Fig 2C, blue line, S1 Movie). Consequently, head and body orientation deviated during head saccades and realigned again during intersaccadic phases (Fig 2C, green line).

### Wing beat kinematics & gaze behavior

Due to their visuomotor capabilities, birds are model organisms in the fields of animal locomotion and visual information processing. However, not much is known about how flight kinematics and visual behaviors are tuned to work in concert. When analyzing wingbeat kinematics and gaze behavior across all five birds we found that: (i) the wingbeat of lovebirds is intermittent during maneuvering flight, and (ii) saccadic gaze shifts are preferably initiated at the end of the downstroke.

#### Wingbeat & flight kinematics

We analyzed the wingbeat kinematic data with a Gaussian mixture model to determine if they are bi-modally distributed due to intermittency. The resulting Gaussian fits reveal that both the flapping frequency and the relative periods of the down- and upstrokes are bimodal distributed for each individual bird. Lovebirds almost halved their normal flapping frequency (17.01 Hz ± 0.87 Hz, mean ± S.D.) during intermittent flaps (9.58 Hz ± 0.48 Hz, mean ± S.D.; Eq 1; Fig 3A). They accomplished this frequency reduction by extending the upstroke duration to twice the time of the downstroke. During an intermittent flap, the ratio of down- to upstroke period was 0.53 ± 0.06 and the wingbeat was performed at about 9.5 Hz. In contrast, normal wingbeats were performed at approximately 17 Hz, and the down- to upstroke ratio averaged at 1.35 ± 0.11. The downstroke is thus 35% longer than the upstroke. To see whether the flapping intermittency is modulated between phases of the U-turn, we analyzed the percentage of intermittent *vs*. normal frequency wingbeats in each phase, for each individual bird, and averaged the results. There is no general difference in intermittency between phases (p = 0.074, Friedman test with Bonferroni correction, *n* = 697 wingbeats) but intermittent flaps (*n* = 153) tended to happen most often in the phase after the turn, while normal flaps (*n* = 528) tended to occur predominantly during the turn. Of all recorded intermittent flaps, 26.5% ± 12% occurred in the phase before the turn, 29.8% ± 9.9% during the turn and 43.7% ± 13.14% after the turn across birds. Of all normal flaps 30.8% ± 5% occurred before the turn, 40.9% ± 5.2% during and 28.7% ± 3.6% after the turn.

To see if flight speed varied between flight phases, we calculated the average flight speed per wingbeat and averaged it for each bird over each flight phase. Lovebirds flew 0.54 m/s ± 0.01 m/s slower during the turning phase than during the phase before the turn (1.05 m/s ± 0.16 m/s) and the phase after the turn (1.03 m/s ± 0.16 m/s; p = 0.02, Friedman test with Bonferroni correction, *n* = 697 wingbeats). The advance ratio J, is a non-dimensional parameter defined as the ratio between forwards flight speed and the mean wingtip velocity (Eq 2), it indicates if an animal is in slow hovering flight or forward flight [26]. In accordance to the wingbeat average flight speed, the advance ratio was significantly reduced in the turning phase (0.056 ± 0.01) as compared to the straight flight phase before the turn (0.12 ± 0.03) and the straight flight phase after the turn (0.11 ± 0.01; p = 0.02, Friedman test with Bonferroni correction, *n* = 697 wingbeats). This quantitative result confirms the general observation that rapid turning lovebirds were in slow-hovering flight throughout the maneuver [26].

#### Coordination of gaze with wingbeat kinematics

Are gaze shifts in the form of superfast head saccades related to wingbeat kinematics? As apparent from scrutinizing the saccadic gaze shift timing in the individual example (Fig 2D, black trace, S1 Movie), the initiation of a head saccade was performed specifically during downstroke phases of the wingbeat. To analyze this observation more quantitatively, we noted the relative point in time of saccade initiation for the downstroke and for the upstroke phases of a wingbeat. From the 16 flights in which we analyzed head motion at 2000 Hz, 66 of 72 detected head saccades were made during the downstroke (Fig 3B, green distribution). Saccade probability was highest in the last third of the downstroke. Only six saccades were made during the upstroke phase (right after the start). The duration of a saccade was typically half a wingbeat (see head kinematics section below). Saccades that started during the downstroke were, therefore, usually completed during the upstroke (Fig 3B, red distribution). Some saccades lasted into the next wingbeat and ended in the first half of the downstroke. The differing shapes of start and end distributions point out that head saccades were variable, which we show quantitatively below.

### Head saccade kinematics

In contrast to humans, birds shift their gaze mainly through head reorientation (review: [14]). Head saccades can therefore be analyzed to assess gaze performance and strategies. We found that lovebirds performed head saccades more frequently and faster during turning flight. Combined, head saccades made up 76% of the total head reorientation during the turn. Saccade velocities reached values of up to 2700 °/s and are thus, in terms of speed, comparable to head saccades in insects which are three orders of magnitude lighter (Fig 4). Although the head saccade amplitudes are comparable with eye saccade amplitudes of humans and rabbits, lovebirds perform head saccades about three times faster (Fig 5; based on time-resolved tracking data at 2000 Hz). When plotting saccade start position over the flight trajectory, we found that lovebirds make most saccades during the turning phase (Fig 4A, circular markers). Saccades during the turn were typically faster than saccades before or after the turn (see color code in Fig 4A). To compare saccade parameters between birds, we averaged the saccades over individual flights for each bird, before calculating the average across birds. In general, head yaw saccades reached amplitudes up to 60° with a median of 27.7° across birds (Fig 4B). Remarkably, head saccade amplitudes with respect to the arena were comparable to head saccade amplitudes with respect to body yaw orientation (p = 0.96: Friedman test with Bonferroni correction). This shows that head saccade amplitude was not substantially modified by body rotation. Saccade duration varied and had a median at 29.6 ms. The longest recorded saccade lasted 44.5 ms (Fig 4C). While the median head saccade velocity was 926.1°/s across turning birds, rotational head velocities were dramatically reduced during intersaccades to 146.4°/s (Fig 4D). We quantified head yaw velocities relative to the arena and relative to the body (Fig 2C, green line) and averaged them over both the saccadic and intersaccadic phases (Fig 4E). Head yaw velocities with respect to the body were higher between head saccades than absolute yaw velocities with respect to the arena (compare red distributions Fig 4D and 4E; p = 0.03, Friedman test). This shows the extent to which the head was stabilized during intersaccades. In contrast, during head saccades, the head yaw velocity with respect to the body was lower than the yaw velocity with respect to the arena, because the head and body turned in the same direction (compare blue distributions Fig 4D and 4E; p = 0.025, Friedman test). When plotting the distribution of peak saccade velocities with respect to the arena it becomes obvious that most head saccades exceeded velocities over 1000°/s (median 1290°/s). The highest measured head saccade velocity was 2700°/s. This value reaches almost head turning speeds achieved by flying blowflies [27], 1000 times lighter than lovebirds, and it exceeds head turn speeds reported for honey bees [28]. This is even more remarkable considering lovebirds are four times heavier than zebra finches, which they also outperform [3]. The fastest in-flight saccade speed has been reported for fruitflies, performing saccadic body turns which reach 5400°/s during escape maneuvers [29]. Whereas these metrics are impressive, we wondered how saccade amplitude and duration compares across vertebrates.

To determine how saccade amplitude is related to its duration, we plotted the saccade amplitude as a function of the saccade duration (Fig 5A). We found a strong correlation between the amplitude and the duration of a saccade (r = 0.88). The data can be approximated with a linear regression (y = 1.5*x-14, slope = 1500°/s, R^2^ = 0.78), suggesting that lovebirds have a preferred average head saccade velocity of 1500°/s. To evaluate this finding in a broader context, we extracted saccade amplitude and duration data from figures of earlier studies investigating gaze behavior in visual model organisms, specifically vertebrates (Fig 5A, [30–33]; the open source software ImageJ was used for data extraction http://imagej.nih.gov/ij/index.html). Compared to the combined eye-head gaze shifts of rhesus monkeys, and the eye saccades of humans, rabbits and cats, the saccadic gaze shifts of maneuvering lovebirds are extraordinarily fast. There is a general trend that saccades in lovebirds are shorter than in others species, although their amplitudes are comparable to eye saccades in humans and rabbits. Horizontal head saccades in lovebirds are much larger than eye saccades of the cat. Combined gaze shifts in rhesus monkeys (head + eye) reach higher amplitudes than lovebird head saccades. The variability in saccade amplitude stimulated us to determine how much saccadic *vs*. intersaccadic gaze changes contribute to the total orientation of the head during a U-Turn. Hence, we summed all saccade amplitudes and compared it to the total head reorientation during the turn. We found that, on average, three quarters of the horizontal head reorientation was accomplished through saccadic gaze changes (76.25% ± 3%; Fig 5B). This value was similar in all birds (p = 0.62, Kruskal-Wallis test), showing that gaze changes were predominantly facilitated by super-fast head saccades, but towards which arena features?

### Gaze orientation during flight

Due to their highly flexible necks and relatively light heads, birds facilitate large gaze shifts primarily by fast head turns. The fast head saccades we report here can be considered as fast gaze shifts, because the relative eye movements in unrestrained birds are less than about 10° [14]. Like in humans, visual perception in birds is suppressed during fast head and eye saccades [34,35]. Hence we wondered which features lovebirds keep stabilized in their visual field between saccades.

By combining head orientation and flight arena geometry (Fig 6A), we found that maneuvering lovebirds stabilize high contrast features in their frontal visual field during intersaccades. Therefore, we indicate the range of feasible eye movements, which results in an uncertainty in the estimated gaze direction in the frontal visual field, using a gray horizontal bar in Fig 6B and Fig 6C–6K. Typical gaze shifts (based on Fig 2) are shown in Fig 6B. To analyze gaze distributions quantitatively, we averaged retinal feature positions across birds (*n* = 92, *N* = 5) [21]. Due to motion blur [36] and saccadic suppression [34,35], we focused our quantitative gaze analysis on intersaccadic phases for which head orientation is relatively constant (Figs 2D and 4D). We investigated these phases across 92 left turn flights (including the 15 left turns resolved at 2000 Hz) for which we then tracked the head at four times the wingbeat frequency (about 68 Hz). To separate saccadic from intersaccadic phases in this larger data set, Fig 4A, we excluded head yaw data which exceeded a standard deviation of 10° yaw per wingbeat (± 10°: gray shaded bar in Fig 6B–6K, [13–15]). This value is based on the largest horizontal eye movements that may assist in stabilizing gaze. Azimuthal angle distributions were independent of acquisition rate (correlation coefficients for compared arena features > 0.9). Hence, lower temporal resolution wingbeat data can be used robustly to determine intersaccadic head orientation.

During the flight phase before the left turn, the lovebirds’ head was directed at the center of the high contrast corner, formed by the white sidewall and the dark headwall. This high contrast edge was thus centered in the middle of their visual field (Fig 6A and 6B violet line; Fig 6C dotted line). During the left turn, lovebirds shifted the relative position of arena features multiple times to the right by saccadic head turns to the left (Fig 6B). The magnitude of these shifts depends on the amplitude of the yaw saccades (compare Fig 6B with 2D). While turning, the majority of birds first centered the left high-contrast edge of the white sidewall (Fig 6A and 6B light green line; Fig 6D). During intersaccades they faced the right edge of the perch (Fig 6A and 6B dark green line; Fig 6E). After turning, the majority of flights ended with a saccadic gaze shift to center the perch and stabilized it frontally (Fig 6A and 6B green line; Fig 6F). As a consequence of frontal feature stabilization (Fig 6G–6K), other adjacent features have lower laterally positioned probability peaks (compare after turn distributions of Fig 6F with Fig 6E and 6K). For the above described gaze analysis, the position of the perch in rest was used, despite the fact that the perch was swinging forward and backward (amplitude ~10 cm; sideward amplitude ~1 cm). We checked whether this perch motion changes our conclusions, by calculating the actual perch azimuthal position with respect to the bird for the higher resolved data set (*n* = 15 left turn flights, *N* = 5). We found that the results were quantitatively similar for a static and a moving perch (dashed lines in Fig 6E, 6F and 6K). Birds encounter moving perches in their environment when landing on swaying branches and power lines in wind, so which visual cues do birds use to coordinate landing on moving perches?

### Visual cues for coordinating landings on a swinging perch

Studies in the past have shown that birds and insects use visual cues during the landing approach, such as the azimuthal retinal extent of the landing site (α), its relative retinal expansion velocity (RREV), and the estimated time-to-collision (tau), to initiate landing behavior on a static spot (houseflies: [37,38]; fruitflies: [39]; bees: [40]; pigeons: [4,5]; hawk: [5]). To obtain the RREV and tau birds rely on the experienced α and its rate of change over time, the experienced retinal expansion speed (Ω). We obtained α by calculating the difference angle between the azimuthal angles for the right and left perch edges for every recorded frame (Fig 6A). We estimated Ω from taking the derivative of α. We calculated RREV as the ratio Ω/α and tau as the ratio α/Ω [39].

To evaluate which of the mentioned parameters are likely used by lovebirds to control landing on a swinging perch, we applied a coefficient of variance (c.v.) analysis introduced by Wagner for landing houseflies [37] and subsequently used for landing pigeons and a hawk by Green and Davies [5]. The general assumption is that the parameter varying least across individuals and trials before landing initiation is the parameter most likely (parsimonious) to trigger landing behavior. The c.v. for each optical parameter was calculated by dividing their standard deviation across trials by their means across trials, for each time step before landing initiation. In our case, we defined the point in time of downward pitch of the tail feathers as the indicator for landing initiation. Bird’s visuomotor delay is in the range of 30 ms to 70 ms [41,42], we therefore started analyzing control parameters 85 ms before the down pitch of the tail feathers.

The dynamic swinging motion of the perch induced large variations in the relative approach speed (compare Fig 7A & 7B). We found that in all cases α varied least (Fig 7C). This low variation indicates that α is the most parsimonious to trigger landing behavior in lovebirds landing on a moving perch, such as a branch swaying in the wind.

## Discussion and Conclusions

**Fig 8:**
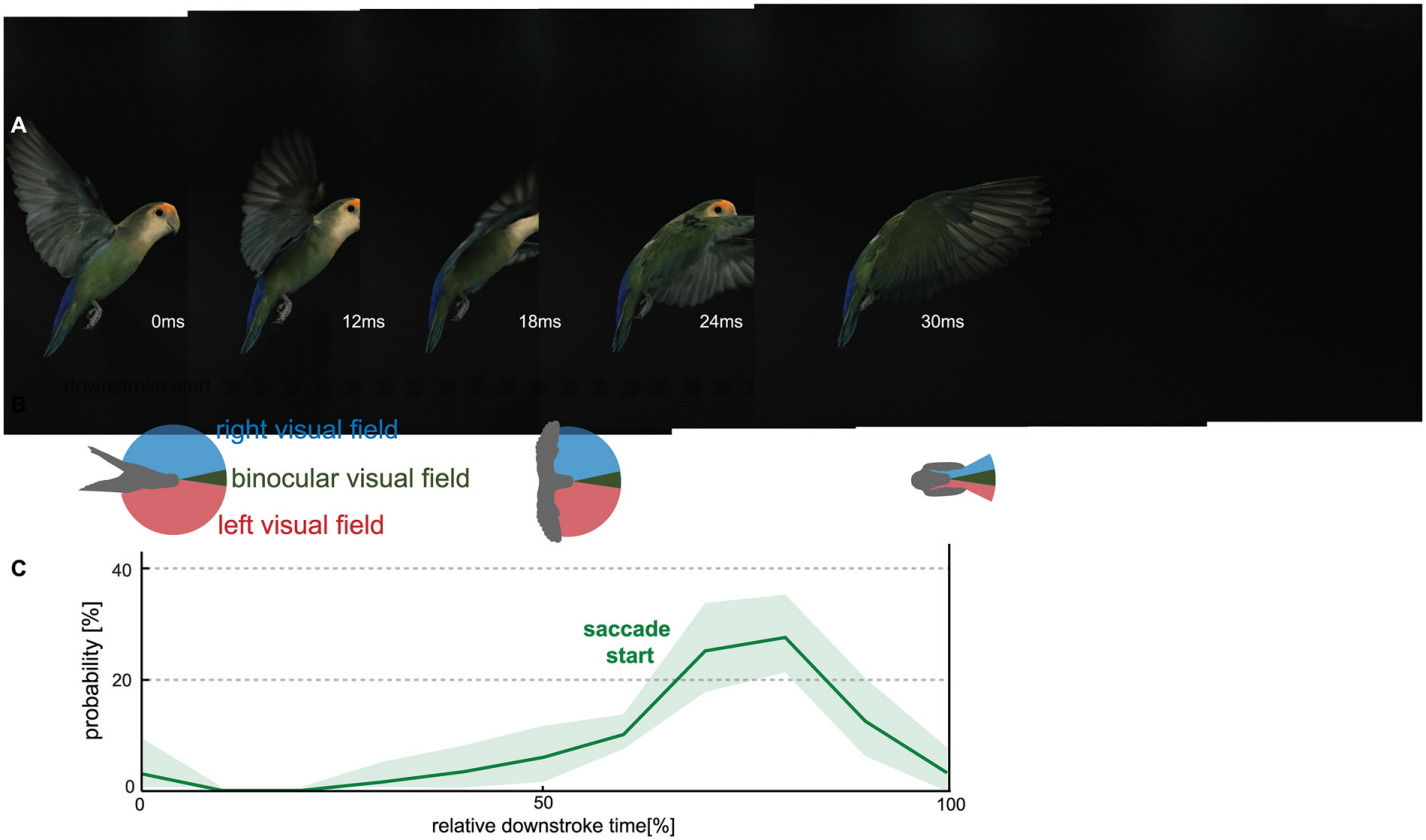
Lovebirds improve visual flight control by coordinating super-fast gaze shifts with the end of their downstroke. (A) Top view schematic of a typical recording that shows that the wings occlude the lateral visual field at the end of the downstroke. The lovebird’s azimuthal visual field is approximated from ophthalmologic measurements at the visual equator of Senegal parrots [57]. (B) For demonstrative purposes a lovebird was filmed from the side during a turning on a dime maneuver with the side panels of the arena removed (this video sequence is not part of the data analysis). (C) Most saccades were initiated at 75% of the downstroke (see Fig 3B), when the wings occlude more than half of the lateral visual field.

Lovebirds performing a rapid ‘turn on a dime’ maneuver shift their gaze between arena features with superfast head saccades. These gaze shifts reach on average 930°/s with peaks up to 2690°/s, the fastest recorded saccades for vertebrates to date [14,36] (Fig 5A). This speed compares to the saccade speeds reported for flying insects that are up to three orders of magnitudes lighter than lovebirds [27] (Fig 4F). These extraordinarily fast turns are made possible by the highly specialized avian neck system, of which the muscles contract effectively as fast as the flight muscles [43]. Between saccades, lovebirds stabilize their head towards prominent arena features in the frontal visual field (Fig 6). Compared to the head, body rotations are continuous and slower, lacking the characteristics of saccades (Fig 2C, S1 Movie). This finding indicates that flying lovebirds perform a gaze strategy that facilitates optic flow processing [3,44,45]: They are separating distance dependent translational optic flow from distant independent rotational optic flow cues at the behavioral level. This optic flow orchestration simplifies neural processing [8,45] in visual brain centers [46–50], which facilitates the extraction of relative proximity and heading information. A similar head turn behavior has been reported for turning pigeons [51]. However, due to the different focus of their study, Bilo and colleagues interpreted their findings mainly within the framework of reflexive neck and wing control mechanisms [51]. Our finding that lovebirds landing on a moving perch experience low variation in retinal perch size, supports the hypothesis that landing birds use this cue for visual flight control, in addition to self-motion related parameters [5]. Lovebirds shift their gaze between arena features using super-fast saccades that are timed with the last quarter of the wings’ downstroke (Fig 3B). As their wings occlude lateral vision during this stroke phase, lovebirds maximize visual perception by overlying behaviors that impair vision (Fig 8). In flying bats a similar coupling between wing kinematics and acoustic sensing was found. Their respiration and ultrasonic calls are related to their wingbeat frequency [52–54]. By analyzing the wingbeat frequency of maneuvering lovebirds, we find them to be intermittent as reported for other small generalist birds [55,56]. Interestingly, lovebirds compose their wingbeat of two distinct flapping modes (Fig 3A). During their slower flapping mode of 9.5 Hz, upstrokes are twice as long as downstrokes while downstrokes are slightly longer in the normal flapping mode of 17 Hz. When turning, lovebirds perform preferably normal wingbeats and, accordingly, most observed saccadic gaze shift are coordinated within this flapping mode.

### Super-fast gaze changes

When comparing lovebird saccadic gaze shifts with the ones reported for other vertebrates, it becomes apparent that maneuvering lovebirds shift their gaze faster than other animals serving as visual model systems (vertebrate review: [14], cat: [32], rabbit: [33] chameleon: [58], goldfish: [59], frog: [60], zebrafish: [61], primates: [62]) (Fig 5A). For example, visually induced gaze shifts in humans, which are among the fastest in vertebrates, reach up to 500°/s when head and eye movements are combined [63] and around 400°/s when only the eyes are moved [31]. Lovebirds are on average twice as fast at about 930°/s. Fast gaze shifts reduce the time during which visual information processing might be impaired due to motion blur and, thus, prolong the net time during which visual information can be obtained. The fact that birds also have relatively fast flicker fusion frequencies above 100 Hz, and thus, a higher motion sensitivity compared to other vertebrates [64,65], further shows that birds have evolved extraordinary visual capabilities. This high-performance visual and gaze control is essential to guide fast flight through cluttered habitats.

### Control of head-neck motion during saccadic and intersaccadic phases

The rapid horizontal gaze shifts and the gaze stabilization between saccades are facilitated by the sophisticated avian head-neck system and its sensory motor control [66]. The avian neck is built up by more than twice as many vertebrae compared to a mammal, and is actuated by over 200 muscles on each side. These muscles can be classified in five functional groups from which the first, the carnio-cervical and the fourth, the lateral system appear to be most relevant for horizontal head rotations relative to the body [43]. Both muscle groups contain strongly pinnate mono-articular muscles actuating motion around several vertebral joints at once. Their muscle fibers are very long which allows especially fast contractions [43,67,68]. This specialized musculoskeletal system and the relatively light head enables birds to make rapid precisely coordinated head turns. Indeed, the median saccade duration in lovebirds is just 29.6 ms, which corresponds to a contraction time as fast as flight muscle contraction (a lovebird downstroke takes about 30 ms).

#### Saccadic head turn

How may the here reported saccadic head turns be controlled? There are two well-studied gaze behaviors that induce rapid head turns in space. The first behavior is the reflexive fast phase of the optocollic reflex (OCR), of which the function is interpreted to be similar to the fast resetting of the eyes; the optokinetic nystagmus (OKN). Brain systems involved in the control of both reflexes are the nucleus of the basal optic roots (nBOR), the pretectal nucleus lentiformis mesencephalic (LM) and the vestibule-cerebellum [69]. The second behavior facilitates voluntary saccadic gaze changes towards a visual feature [14,66]. As the LM and nBOR are involved in controlling horizontal eye saccades [70,71], they are also likely candidates to control visually induced horizontal head saccades. The head-body relation we observed (i.e. Fig 2B green line) are slightly different from the classical OCR response observed in restrained animals that stabilize a moving pattern [72]. Although, head and body turn in the same direction during the turn on a dime maneuver, turning lovebirds increase the deviation angle between head and body with their saccades. This observation is similar to head motions in maneuvering zebra finches [3] and blowflies [27]. In contrast, body-restrained birds make saccades directed to the body to re-center their head orientation while stabilizing a pattern on their retina [72]. Our observation that lovebirds turn their head from normal orientation towards salient visual features, such as the perch edge and its center, indicates that a visual function is most parsimonious (example saccades 2 and 3 in Fig 2E and quantitative results in Fig 6). Similar saccadic eye-in-head traces have been described for primates performing a series of eye saccades during a visual fixation task with continuous head rotation (Fig 6 in [62]).

#### Head stabilization between saccades

Between saccadic gaze changes, rotational head stabilization in space is likely coordinated by the vestibulo-collic reflex (VCR) [62,66]. Visual control in the form of the optocollic reflex (OCR) may increase rotational head stability between saccades even further. In addition to possible sensory functions, the reflexive head stabilization in space promotes head-body realignment, which is mediated by the continuously turning body. Interestingly, the velocity tuning of the OCR and VCR change with the activity state of birds [73–76]. The exact roles and integration of the vestibular and visual system during free flight are, however, not well understood.

### Gaze approximation based on head orientation

To understand how the observed gaze behavior is used to fixate image features we need information about head stabilization, eye motion, and the actual focus of visual attention on the retina. So far only head orientation data has been obtained in freely flying birds [3,10,44,51,77–80], eye orientation and/or focus of attention have only been measured in head fixed [1,57,81–83], body restrained [15,84], and walking [85] birds. To interpret how the observed head stabilization behaviors in free flight might facilitate image stabilization we assume eye motion is small in flight. This is supported by the observation that birds predominately change gaze by turning their heads (review [14]). In contrast, humans prefer to facilitate gaze shifts of up to 45° with eye movements [63], which are of similar amplitude as the head saccades we report here for lovebirds. In head unrestrained pigeons, gaze stabilization to horizontal wide field motion is facilitated by a combination of optokinetic and optocollic reflexes. Image stabilizing head rotations account for 80–90% of the total gaze shifts. However, when restraining the head, eye movements rose to 90% of the total gaze shift [13,86], this supports our assumption that head-free birds coordinate gaze orientation through head saccades. Indeed, studies quantifying the amount of eye movements in unrestrained pigeons, chickens, and peahens found that horizontal eye movements are typically below 10°, much smaller than the anatomical range [15,16,85].

Whereas we can assume that eye orientation is coordinated with head saccades in free flight, we do not have retinal morphological data to determine how head orientation relates to fovea orientation. Temporal regions with higher visual acuity that are frontally oriented like in cockatoos [87] seem to be absent in parrots [57,88]. However, the role of foveation in flight has not been established. Thus, free flight foveation experiments are needed to assess if maneuvering birds fixate salient visual features frontally with their retinal regions of high visual acuity.

### Which visual parameters help guide landing on a swinging perch?

By scrutinizing the high-speed flight recordings, we found that the first behavior indicating landing is a downward pitch of the tail feathers that resulted in an upward pitched body posture accompanied by an extension of the legs. Therefore, and because the exact timing of leg extension was difficult to assess from our videos, we based our definition of landing initiation on the tail pitch behavior. The tail pitch analysis starts up to 50–100 ms earlier than the previous analysis based on leg extension ([4,5]; we compared tail pitch and visible leg motion timing in our recordings). We find that retinal size is most parsimonious to initiate a downward tail pitch in lovebirds when they land on a swinging perch, as has been proposed for pigeons landing on a static perch [5]. For a perch of known size, its retinal expansion can be a direct measure for its distance. We did not observe head-bobbing behavior as reported for landing pigeons [79]. In contrast to pigeons and insects landing on a plane, we found no clear monotonic dependence of flight velocity reduction and retinal expansion speed (correlation coefficient of both parameters: r = 0.58, *n* = 16 flights, *N* = 5), or rate of change of retinal expansion speed (correlation coefficient of both parameters: r = 0.45, *n* = 16 flights, *N* = 5) [5,40]. On some occasions, the relative flight velocity even increased for increasing retinal expansion speed, because the perch started swinging towards the bird (green trace in Fig 7B). To see how the sampling rate of visual cues affected the calculation of temporal derivative based parameters like the retinal expansion speed, RREV, tau and their respective c.v. values, we down sampled our dataset from 2000 Hz to 50 Hz (as used in pigeon studies [4,5]). Interestingly, we found that c.v. values changed minimally but the general order of parameter variability did not change at lower temporal resolutions. Retinal perch size had consistently the lowest c.v. values. However, at both temporal resolutions, we saw a variability drop of RREV around 80 ms before landing. Consequently, our data suggests that retinal size is used as a cue for coordinating the final phase of landing on a moving perch. Further, the data indicates that in early phases before landing the RREV could as well be used as reported for insects [37,39].

### Stabilization of features and high-contrast edges improves optic flow

The finding that lovebirds stabilize wall and perch edges before and during turning, and the perch center after turning to coordinate their landing, may indicate that birds and humans use similar features for navigation. Edges of obstacles that need to be avoided (the arena walls) are visually stabilized by lovebirds, similar to the center of the visual target they choose to approach (the perch) [11]. Such a gaze strategy is thought to facilitate optic flow based navigation [12]. According to the theory, stabilization of obstacle edges results in strong optic flow discontinuities of obstacle and background, which improves experienced visual scene segmentation—a key feature for path planning [12]. Frontal feature stabilization may help birds to reduce distance-independent rotational image flow and, thus, support self-induced motion analysis [49,50,89]. Moreover, the target image expands symmetrically when a visual goal is stabilized in heading direction, which simplifies proximity computation. The amount of image expansion depends on feature distance and approach speed [7], and might therefore be involved in the control of the goal approach—landing in our case. This theory is supported by our landing analysis. We found that 30 ms to 85 ms before landing initiation (downward tail pitch) the perch size (α) varied least. In addition, relative retinal expansion velocity (RREV) varied second least. Consequently, both variables are plausible factors for initiating landing [5,37,39]. The size of the swinging perch (α) is, however, the most parsimonious control parameter in our study because it varied least.

### Tuned gaze shifts for increased visual awareness

Flapping animals frequently conduct subtle adjustments to their wing movements, body posture, and head orientation to stabilize themselves [90]. Our observation that lovebirds time the start of the saccade at 75% of the downstroke can be understood as a strategy to maximize visual awareness. At 75% of the downstroke the wings are in a position lateral to the head and occlude the lateral field of view (Fig 8); lovebirds thus facilitate visual perception by overlying behaviors that impair vision. This finding complements the reports that birds are specifically motion sensitive in the lateral field of view [91] and depend on this input for visuomotor control [2]. The minimal delay associated with avian visuomotor control is about 30 ms [42]. Considering the birds’ wingbeat lasts 60 ms and that saccades were initiated at about 23 ms into the downstroke, it is unlikely that visual information obtained during the same downstroke triggers the saccadic gaze shift. Instead, the most parsimonious interpretation of the gaze-shift behavior is that translational image motion obtained in the previous wingbeat(s) triggers the saccade. This has been found for insects during collision avoidance [92–94]. For insects it is, however, unknown if they time their saccade as exquisitely within a wingbeat as lovebirds do. In contrast to the head saccade, we found that the tail pitch is not precisely coordinated with the wingbeat. This emphasizes that saccadic gaze changes are precisely tuned with the wingbeat, whereas some other motor commands related to flight control are not. The super-fast gaze behaviors we describe here for lovebirds can aid vision-based autopilots for robots flying in cluttered and GPS-denied environments.

## Supporting Information

S1 Movie Slow motion video of a lovebird performing a turning on a dime maneuver. The bird performed a saccadic gaze behavior timed within the wingbeat. Between saccadic turns, the head is rotationally stabilized. In contrast, the body turns continuously during the maneuver following the head. The bottom graph illustrates the estimated head and body yaw orientation relative to a horizontal in the arena. An orientation of 0° represents a straight flight away from the perch, 180° a flight to the right towards the perch. Vertical bars indicate the downstroke phases and thereby the wing beat timing. The video is 50x slowed down for illustration. The actual maneuver took 1.15 seconds. See Fig 2 for more quantitative results.
(MP4)
