# Correction: How Lovebirds Maneuver Rapidly Using Super-Fast Head Saccades and Image Feature Stabilization

**DOI:** 10.1371/journal.pone.0133341

**Published:** 2015-07-24

**Authors:** The PLOS ONE Staff

Fig 8 is incorrectly cropped due to errors introduced during the typesetting process. The publisher apologizes for the error. Please see the corrected Fig 8 here.

**Fig 8:**
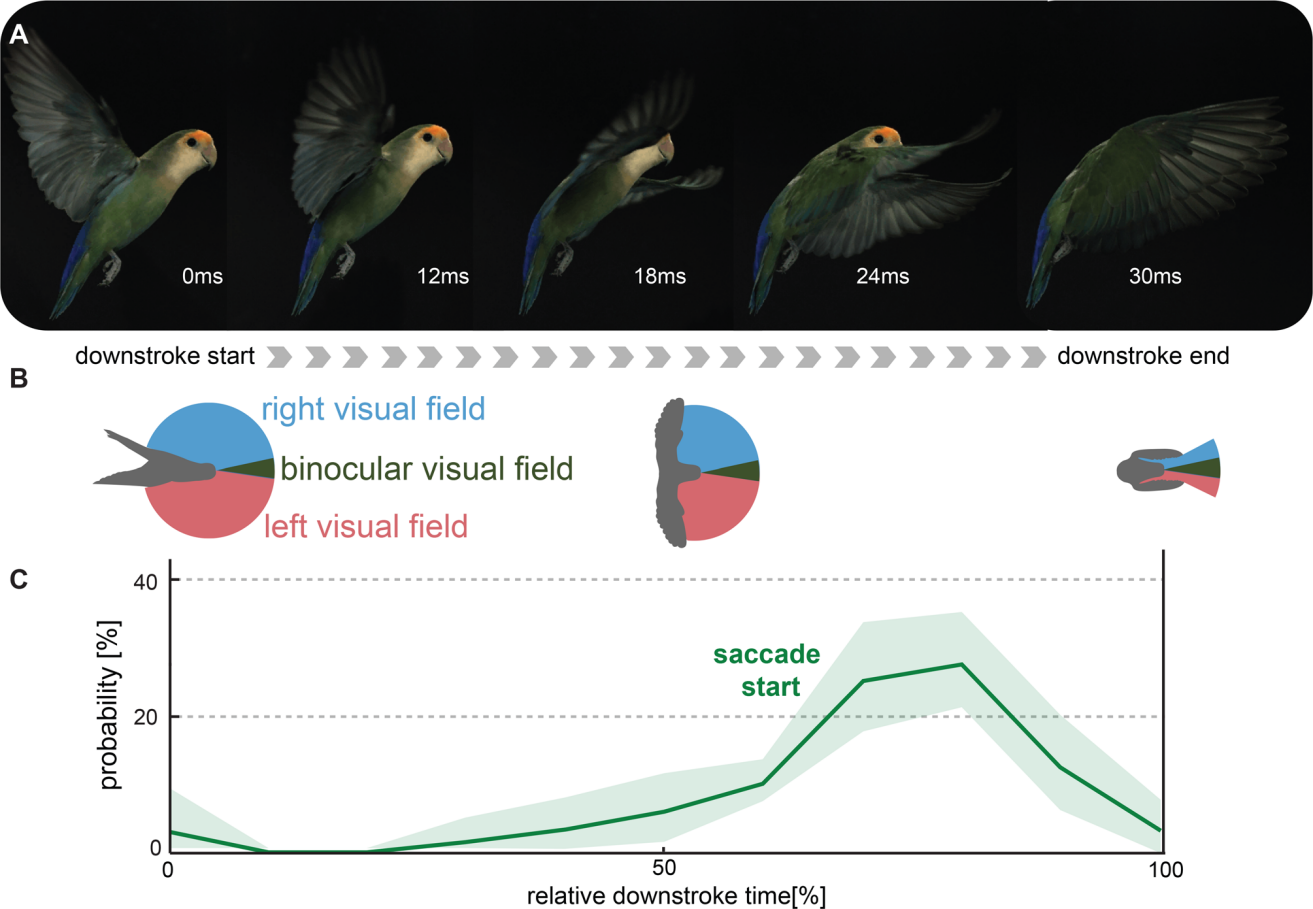
Lovebirds improve visual flight control by coordinating super-fast gaze shifts with the end of their downstroke. (A) Top view schematic of a typical recording that shows that the wings occlude the lateral visual field at the end of the downstroke. The lovebird’s azimuthal visual field is approximated from ophthalmologic measurements at the visual equator of Senegal parrots [57]. (B) For demonstrative purposes a lovebird was filmed from the side during a turning on a dime maneuver with the side panels of the arena removed (this video sequence is not part of the data analysis). (C) Most saccades were initiated at 75% of the downstroke (see Fig 3B), when the wings occlude more than half of the lateral visual field.
